# Pathologic Complete Response and Long-Term Survival After Preoperative Chemotherapy for Transverse Colon Cancer With Para-Aortic Lymph Node Metastases

**DOI:** 10.7759/cureus.59363

**Published:** 2024-04-30

**Authors:** Marina Jimba, Kentaro Nakajima, Masashi Momiyama, Teppei Morikawa, Shouichi Satou

**Affiliations:** 1 Surgery, NTT Medical Center Tokyo, Tokyo, JPN; 2 Diagnostic Pathology, NTT Medical Center Tokyo, Tokyo, JPN

**Keywords:** transverse colon cancer, preoperative chemotherapy, para-aortic lymph node metastases, folfox, bevacizumab, case presentation

## Abstract

A 52-year-old male patient was diagnosed with transverse colon cancer and synchronous stage IVA para-aortic lymph node (PALN) metastases (cT3N1bM1a of the lymph node). Six courses of mFOLFOX6 plus bevacizumab were administered as neoadjuvant chemotherapy. Computed tomography showed shrinkage of the primary tumor and PALN metastases. Extended right hemicolectomy, D3 lymph node dissection, and PALN dissection were performed. A pathologic examination indicated that the tumor had completely changed and comprised necrotic tissue with no viable cells. Therefore, it was considered that mFOLFOX6 plus bevacizumab resulted in a pathologic complete response. Postoperatively, six courses of mFOLFOX6 were administered. Six years postoperatively, the patient did not exhibit any signs of recurrence. There have been few reports of pathologic complete response after neoadjuvant therapy and resection for colon cancer with synchronous PALN metastases. This report describes a unique case involving a pathologic complete response with long-term survival after mFOLFOX6 plus bevacizumab and radical resection, including PALN dissection. Preoperative mFOLFOX6 plus bevacizumab followed by radical resection and adjuvant mFOLFOX6 therapy was safe and resulted in a good outcome. This regimen should be considered for advanced colon cancer with PALN metastases.

## Introduction

Colon cancer is one of the leading causes of morbidity and mortality worldwide. Approximately 20% of patients with colorectal cancer have stage IV disease [1]. Extraregional lymph node metastasis is considered a systemic disease, and surgery is a potentially curative option for some patients with limited metastatic disease, particularly for those with disease located in one organ (liver, lung, or others). Recently, advances in chemotherapy and radiation therapy have resulted in their use as potentially curative therapies for patients with isolated para-aortic lymph node (PALN) metastases [2-5]. We report a case of advanced transverse colon cancer with PALN metastases in a patient who underwent preoperative chemotherapy and achieved a pathologic complete response.

## Case presentation

**Figure 1:**
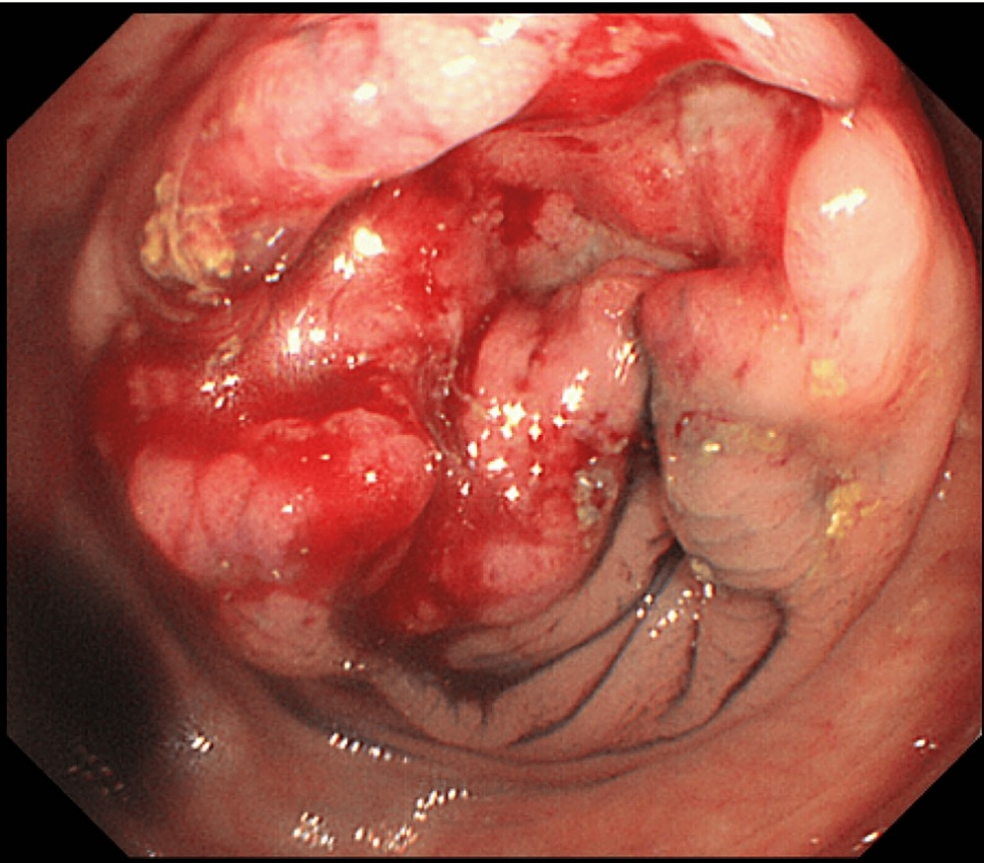
Image obtained during colonoscopy before neoadjuvant chemotherapy: Showing a circular elevated tumor with ulceration in the transverse colon.

A 52-year-old male patient with occult blood in his stool was referred to our hospital for evaluation and treatment. A colonoscopy revealed a type 2 tumor in the transverse colon (Figure 1).

**Figure 2:**
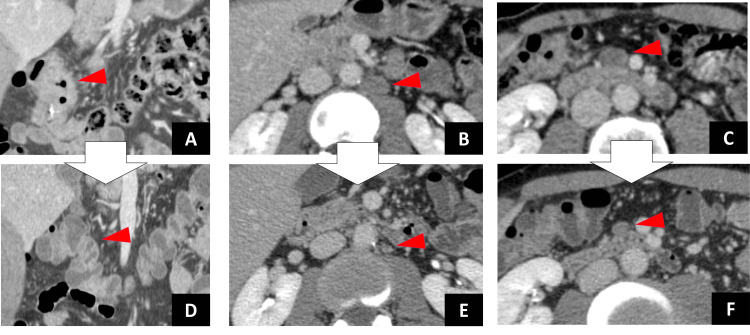
Computed tomography (CT) images. CT images obtained before preoperative chemotherapy: (A) wall thickening at the transverse colon (arrowhead); (B) isolated swollen lymph nodes along the abdominal aorta below the left renal vein (19 mm) (arrowhead); and (C) isolated swollen lymph node (17 mm) (arrowhead). CT images obtained after the administration of six courses of mFOLFOX6 plus bevacizumab: (D) wall thickening not apparent (arrowhead); (E) para-aortic lymph node metastases not apparent (6 mm) (arrowhead); and (F) mesocolon lymph node metastases not apparent (11 mm) (arrowhead).

The biopsy results indicated highly differentiated adenocarcinoma. Genetic analysis revealed the presence of the wild-type *RAS* gene. Abdominal computed tomography revealed wall thickening of the transverse colon, swollen pericolic regional lymph nodes, and isolated swollen lymph nodes along the abdominal aorta below the left renal vein (Figures 2A-2C). Laboratory data revealed high levels of cancer embryonic antigen (CEA) (4.8 ng/mL) and carbohydrate antigen 19-9 (CA19-9) (46 U/mL). The initial diagnosis was stage IVA (T3N1bM1a of the lymph node) according to the Union for International Cancer Control TNM classification of malignant tumors (8th edition) [6].

Chemotherapy was administered before surgery because the colonic stenosis was not severe. The first course consisted of mFOLFOX6, which comprised L-leucovorin 200 mg/m^2 ^administered simultaneously with oxaliplatin 85 mg/m^2^, followed by a 400 mg/m^2^ bolus of fluorouracil (5-FU) on day 1; 5-FU (2,400 mg/m^2^) was infused over 46 hours every two weeks. mFOLFOX6 plus bevacizumab (5 mg/m^2^ on day 1) was administered since the second course. No chemotherapy-related adverse events associated with chemotherapy were observed. After six cycles of chemotherapy, the tumor marker levels decreased (CEA, 2.7 ng/mL; CA19-9, 17 U/mL), and all lymph node metastases were no longer apparent on enhanced computed tomography. These findings indicated were compatible with a complete clinical response according to the Response Evaluation Criteria in Solid Tumors (Figures 2D-2F).

**Figure 3:**
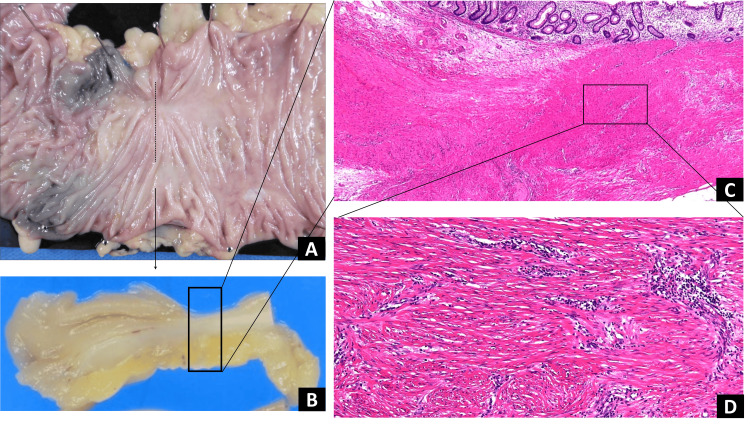
Histological examination of the specimen revealed no viable cancer cells. (A) Fixed specimen of the ascending colon; (B) split specimen; (C) no malignant cells in the colon wall; and (D) fibrosis and lymphocytic infiltration beyond the muscle layer observed.

**Figure 4:**
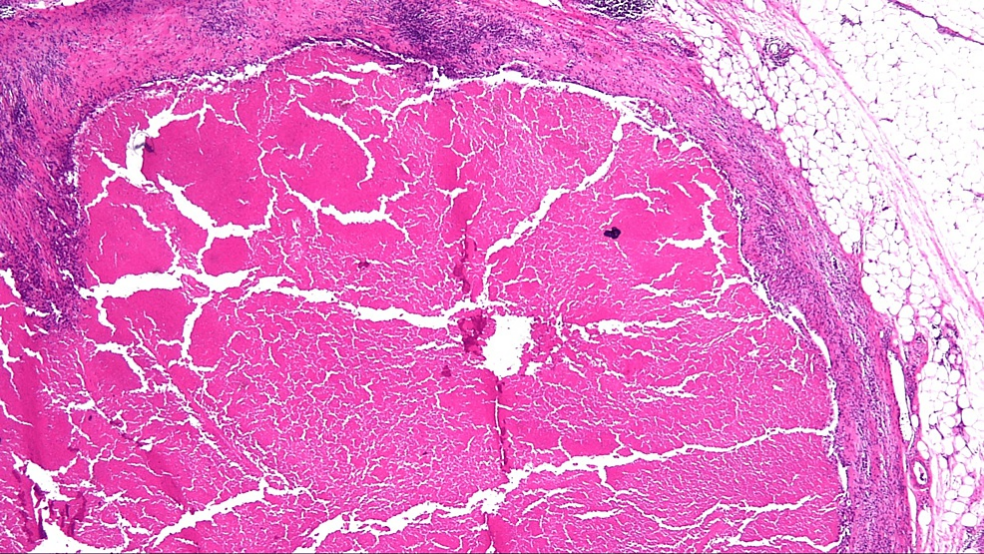
Histological examination of the mesocolon lymph node specimen. Histological examination of the specimen revealed no malignant cells in the mesocolon lymph nodes. Scarring can be observed (×20 magnification).

**Figure 5:**
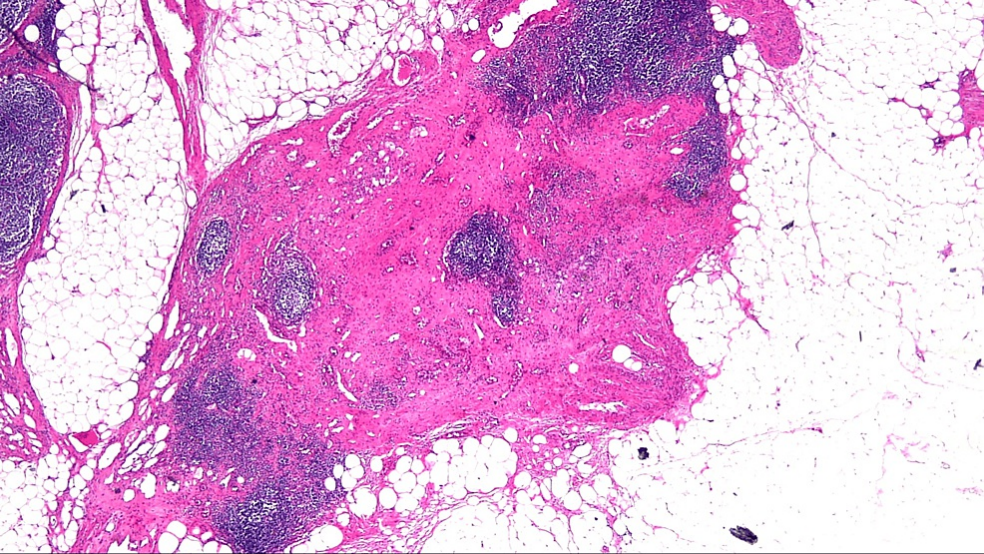
Histological examination of the PALN specimen. Histological examination of the specimen revealed no malignant cells in the para-aortic lymph node (PALN). Scarring can be observed (×20 magnification).

Right hemicolectomy with extensive lymphadenectomy and regional D3 plus infrarenal PALN dissection was performed. Sixteen PALNs were identified. Pathologic specimen examination of the specimens revealed no malignant cells in the colon wall (Figure 3), mesocolonic lymph nodes (Figure 4), or PALNs (Figure 5). Therefore, a pathologic complete response was considered. The patient received six courses of adjuvant mFOLFOX6 postoperatively. At six years postoperatively, the patient did not exhibit any signs of recurrence.

## Discussion

Colorectal cancer is the third most prevalent form of cancer and the second most common cause of cancer-related fatalities worldwide [7]. Before the early 2000s, surgical intervention or a combination of surgery with radiotherapy and/or chemotherapy was the primary treatment modality for metastatic colorectal cancer. However, the advent of targeted medications, such as cetuximab and bevacizumab, resulted in the integration of targeted therapies into clinical management and notably enhanced the overall survival rate of patients [8].

Wong et al. performed a systematic search of the PubMed and Embase databases for isolated PALN metastases in patients with colorectal cancer and found reports of PALN dissections performed in 264 patients with synchronous PALN metastases [9-13]. The PALN dissection procedure described in these reports involved the removal of all lymphatic tissue along the aorta, bounded superiorly by the left renal vein and inferiorly by the bilateral common iliac vessels. No operative mortality was reported. The reported morbidities included acute urinary retention, ileus, surgical site infection, and anastomotic leakage (range 8%-33%). Approximately 19% to 25% of patients received neoadjuvant chemoradiation therapy before PALN dissection [9,11,12]. The five-year disease-free survival rate ranged from 17.6% to 26.5%, and the five-year overall survival rate ranged from 22.7% to 33.9%. The number of positive PALNs obtained intraoperatively ranged from 1.1 to 4.4. The number of positive PALN metastases was identified as a prognostic factor for overall survival [11,12]. A comparison between patients who underwent PALN dissection and those who did not undergo PALN dissection showed that the median survival period was longer in those who underwent PALN dissection (34-40 months vs. 3-14 months) [13]. Wong et al. concluded that resection is a reasonable treatment option for patients with isolated retroperitoneal nodal metastases without progression after systemic chemotherapy and for those with extraretroperitoneal metastases. Eligible candidates for this treatment included those with a primary tumor smaller than 5 cm and fewer than two PALN metastases.

The role of neoadjuvant chemotherapy in locally advanced colorectal cancer remains unclear. The FOXTROT trial, presented at the American Society of Clinical Oncology in June 2019, showed that 59% of patients, including some patients with pathologic complete responses, experienced better surgical outcomes and histological regression after neoadjuvant chemotherapy [14]. However, these survival benefits were not statistically significant during early follow-up. In 2020, Cheng et al. conducted a systematic review and reported that neoadjuvant chemotherapy, compared with adjuvant chemotherapy, exhibited potential survival benefits for locally advanced colon cancer may have a survival benefit without an increase in surgical morbidity. In addition, they found that neoadjuvant therapy regimens resulted in statistically significant improvements in overall survival (hazard ratio [HR], 0.76; 95% confidence interval [CI], 0.65-0.89; *P *= 0.0005) and disease-free survival (HR, 0.74; 95% CI, 0.58-0.95; *P* = 0.02). In addition, the R0 resection rate was not significant (odds ratio, 1.86; 95% CI, 0.95-3.62; *P *= 0.07), and the risk of perioperative complications did not differ between groups. Therefore, they concluded that neoadjuvant or perioperative approaches may be a suitable alternative to upfront surgery followed by chemotherapy for the treatment of locally advanced colon cancer [15]. However, the significance of neoadjuvant chemotherapy in colorectal cancer with isolated synchronous extraregional lymph node metastasis has not yet been established. Ogura et al. reported that 25% of patients received neoadjuvant chemotherapy before PALN dissection; however, they could not clarify its efficacy because of their small sample size and selection bias [11].

Our patient achieved a pathologic complete response after neoadjuvant chemotherapy. Similar to our case, a patient with colon cancer and PALN metastasis who experienced long-term survival after neoadjuvant chemotherapy followed by radical resection and PALN dissection was reported [16]. Suetsugu et al. treated patients with clinical stage IVA (T3N2bM1a of the PALN) transverse colon cancer with neoadjuvant mFOLFOX6 plus cetuximab (six cycles), followed by right hemicolectomy with D3 lymph node and PALN dissection. Their patients achieved a pathologic complete response and did not experience recurrence during five years postoperatively. Survival after achieving a pathologic complete response due to preoperative chemotherapy for colorectal cancer is uncertain because few such cases have been reported; however, preoperative chemotherapy may offer a curative potential. 

There is no evidence of the effect of adjuvant chemotherapy on extraregional lymph node metastases, including PALN metastases. Various studies have reported the administration of adjuvant therapy after synchronous PALN metastases resection for 53% [9], 70% [17], 94% [11], and 100% [10,12,18] of patients. However, no study has compared the long-term outcomes with or without adjuvant chemotherapy after surgery for PALN metastases. Song et al. recommended that surgical resection of PALN metastases should be followed by systemic chemotherapy to control microscopically disseminated cancer cells [12].

## Conclusions

The significance of neoadjuvant chemotherapy for colon cancer with isolated synchronous extraregional lymph node metastasis has not yet been established. Our report presents the case of a patient with colon cancer and PALN metastasis who experienced long-term survival after neoadjuvant chemotherapy followed by radical resection and PALN dissection. Radical resection after preoperative mFOLFOX6 plus bevacizumab and adjuvant mFOLFOX6 therapy resulted in good outcomes. This treatment option may be appropriate for advanced colon cancer with PALN metastasis.
